# Examining the Relationship Between Illness Acceptance, Stigma, and Social Support in Mental Health Care Utilization for Cancer Patients in Hungary

**DOI:** 10.1192/j.eurpsy.2026.11555

**Published:** 2026-07-31

**Authors:** R. Chowdhry, D. Őri, A. Kegye, E. Földesi, V. K. Bognár, L. Losonczy, E. Szenczi-Pinter, A. Guttengeber, A. Petranyi, R. Tóth, G. Purebl

**Affiliations:** 1 Institute of Behavioural Sciences, Semmelweis University, Budapest, Hungary; 2 School of Medicine, University of California, San Francisco, San Francisco, United States; 3 Department of Mental Health, Heim Pal National Pediatric Hospital; 4 Department of Psychiatry and Psychotherapy; 5 Department of Clinical Psychology; 6 Department of Internal Medicine and Hematology; 7 Department of Urology; 8 Institute of Pancreatic Diseases; 9 Department of Obstetrics and Gynecology, Semmelweis University, Budapest, Hungary

## Abstract

**Introduction:**

An understanding of cancer from a psychological perspective is being increasingly prioritized in research and clinical care. Psychiatric disorders have been demonstrated to affect at least 35% and up to 55% of cancer patients throughout their trajectory. Further, cancer treatment adherence and health behaviors have been shown to have a marked decrease due to a series of mental health contributors. Therefore, comprehensive collaborative care interventions incorporating psychological care into oncological care has shown increased promise in reducing the burden of such comorbid diseases and promoting positive health outcomes.

**Objectives:**

We aim to assess perceived stigma towards disease, perceived social support, and acceptance of the illness as related to rates of utilization among cancer patients undergoing treatment.

**Methods:**

We measured the perceived stigma of the disease via the Hungarian version of the Stigma Scale for Chronic Illnesses. The perceived social support was measured by the Hungarian version of the Multidimensional Scale of Perceived Social Support and the acceptance of illness was measured via the Acceptance of Illness Scale and Mini-Mental Adjustment to Cancer to assess coping reactions to cancer. These surveys were administered to 216 cancer patients at Semmelweis University in Budapest, Hungary and data collection took place from November 2024 and is ongoing.

**Results:**

A binary logistic regression was conducted to examine the relationship between the various psychosocial variables and the likelihood of mental health service utilization in the past 12 months for participants until August 2025. The preliminary model was statistically significant, indicating that the predictors were reliable distinguishers between individuals who did and did not utilize mental health care services. The model explained approximately 33% of the variance in service utilization (McFadden’s R² = 0.33), demonstrating a moderate to strong model fit. Among the significant predictors, higher perceived stress (OR = 1.16), anxious preoccupation (OR = 1.17), and stigma-related social constraints (OR = 1.29) were associated with increased odds of service use. In contrast, greater helplessness (OR = 0.80), self-stigma (OR = 0.34), and public stigma (OR = 0.80) were associated with reduced likelihood of using mental health services. Positive help-seeking attitudes were also significant (OR = 1.10), indicating a higher likelihood of utilization.

**Conclusions:**

An in-depth understanding of the social and psychological factors that influence positive health behaviors in the form of mental healthcare service utilization may inform the development of more tailored evidence-based collaborative care interventions.

**Disclosure of Interest:**

None Declared

